# Skin picking disorder in the elderly- What is the available evidence?

**DOI:** 10.1192/j.eurpsy.2024.1310

**Published:** 2024-08-27

**Authors:** O. Vasiliu, A. G. Mangalagiu, B. M. Petrescu, C. A. Candea, C. Tudor, D. Ungureanu, M. Miclos, C. Florescu, A. I. Draghici, R. E. Bratu-Bizic, M. Dobre, A. F. Fainarea, M. C. Patrascu

**Affiliations:** Dr. Carol Davila University Emergency Central Military Hospital, Bucharest, Romania

## Abstract

**Introduction:**

Excoriation disorder (ExD) is a pathology recognized by DSM-5, and it is considered a part of the obsessive-compulsive spectrum. ExD is associated with a high rate of psychiatric comorbidity (e.g., depression, ADHD, substance use disorders, etc.).

**Objectives:**

The main objective of this review was to explore the available evidence to support the diagnosis and treatment of skin picking in elderly population.

**Methods:**

A literature review of the available sources reporting on ExD in elderly patients, realized by searching three electronic databases (PubMed, Cochrane, Clarivate/Web of Science) but also the grey literature. All papers published between January 1990 and July 2023, including the terms “excoriation disorder”, “compulsive skin picking”, “dermatillomania” and “elderly” or “old-age patients” were reviewed.

**Results:**

The information about ExD was extracted almost exclusively from reports on elderly patients with neurocognitive disorders. Tactile hallucinations, delusions of contamination, social isolation and focusing on own bodily sensations, and organic causes- dehydration, allergies, renal insufficiency, hepatic and pancreatic diseases, as well as toxic causes- e.g., adverse events of certain drugs should be investigated in elderly patients exhibiting signs of ExD. A differential diagnosis is very important in this population in order to find the most adequate treatment. Behavioral treatments, serotonergic antidepressants, and glutamatergic modulators have been explored in patients with ExD, although specific trials for elderly patients with this disorder are still lacking. However, case reports support the utility of several serotonergic antidepressants in the elderly.

**Conclusions:**

ExD is a less explored disorder in the elderly, where an extensive differential diagnosis and screening for somatic/psychiatric comorbidities are needed. Trials exploring the potential treatments for ExD in old-age patients are also required for evidence-based case management.

**Disclosure of Interest:**

None Declared

